# Detection of cellular senescence within human invasive breast carcinomas distinguishes different breast tumor subtypes

**DOI:** 10.18632/oncotarget.12432

**Published:** 2016-10-04

**Authors:** Cristina L. Cotarelo, Arno Schad, Charles James Kirkpatrick, Jonathan P. Sleeman, Erik Springer, Marcus Schmidt, Sonja Thaler

**Affiliations:** ^1^ Institute of Pathology, University Medical Center, Johannes Gutenberg University, Mainz, Germany; ^2^ Centre for Biomedicine and Medical Technology Mannheim (CBTM), Medical Faculty Mannheim, University of Heidelberg, Mannheim, Germany; ^3^ KIT Campus Nord, Institute for Toxicology and Genetics, Karlsruhe, Germany; ^4^ Department of Obstetrics and Gynecology, University Medical Center, Johannes Gutenberg University, Mainz, Germany

**Keywords:** cellular senescence, breast cancer subtypes, breast cancer pathology

## Abstract

Oncogene-induced senescence is thought to act as a barrier to tumorigenesis by arresting cells at risk of malignant transformation. Nevertheless, numerous findings suggest that senescent cells may conversely promote tumor progression through the development of the senescence-associated secretome they produce. It is likely that the composition and the physiological consequences mediated by the senescence secretome are dependent on the oncogenes that trigger the senescence program. Breast cancer represents a heterogenous disease that can be divided into breast cancer subtypes due to different subsets of genetic and epigenetic abnormalities. As tumor initiation and progression of these breast cancer subtypes is triggered by diverse oncogenic stimuli, differences in the senescence secretomes within breast tumors might be responsible for tumor initiation, progression, metastasis and therapeutic response. Many studies have addressed the role of senescence as a barrier to tumor progression using murine xenograft models. However, few investigations have been performed to elucidate the degree to which senescent tumor cells are present within untreated human tumors, and if present, whether these senescent tumor cells may play a role in disease progression. In the present study we analysed the appearance of senescent cells within invasive breast cancers. Detection of cellular senescence by the use of SAβ-galactosidase (SAβ-gal) staining within invasive breast carcinoms from 129 untreated patients revealed differences in the amount of SAβ-gal+ tumor cells between breast cancer subtypes. The highest percentages of SAβ-gal+ tumor cells were found in HER2-positive and luminal A breast carcinomas whereas triple negative tumors showed either little or no positivity.

## INTRODUCTION

Cellular senescence is considered to be a pivotal tumor-suppressor mechanism that prevents the outgrowth of oncogenically transformed cells through cell cycle arrest [1, 2]. Oncogenes that have the potential to initiate and promote tumorigenesis generally provoke the induction of cellular senescence to counteract tumor formation. Therefore, a prerequisite for the oncogenic transformation of cells and the subsequent establishment of a lethal tumor is that the cells must circumvent oncogene-induced senescence, and gain the ability to proliferate while expressing activated oncogenes [3]. Studies based on human and murine tissues support the notion that cellular senescence can suppress carcinogenesis [4–9]. The ability of cells to induce the senescence program in response to oncogeneic stimuli depends mainly on the tumor suppressor pathways Arf-p53 and pRB-p16^INK4a^ [10, 1]. Escape from senescence is thought to occur when senescent cells acquire additional genetic or epigenetic alterations that reverse their growth arrest. Thus defects in Arf-p53 and pRB-p16^INK4a^ circumvent the induction of senescence and enhance susceptibility to cancer progression [11–13, 6].

Although escape from senescence is thought to be a central requirement for tumor initiation and progression, senescence can still be induced in tumor cells under certain circumstances [2]. Thus tumor cells may enter senescence in response to conventional anti-cancer therapies such as chemotherapy [13, 14], after reactivation of p53 [15, 16] or through an Arf-p53-independent mechanism based on the loss of Skp2 [17]. In these cases, induction of senescence is associated with tumor regression and the initiation of an inflammatory response that stimulates immune cells to eliminate the senescent cells [16, 18, 19].

Senescent cells develop a senescence-associated secretory phenotype (SASP) in which a variety of factors termed the senescence messaging secretome (SMS) are secreted that can affect the behavior of neighboring and immune cells [1, 20]. Many SASP components are pro-tumorigenic growth factors, proteases or cytokines known to stimulate the aggressive behaviour of cancer cells *in vitro* [21–23]. In mouse xenografts, senescent cells have been shown to promote malignant progression of precancerous, as well as established cancer cells [24, 21]. Additionally, it was reported that the SASP of senescent cells transformed by constitutive HER2 signalling inhibits the clearance of senescent cells and exerts pro-metastatic effects leading to breast cancer progression [25].

Although the paracrine activities of SASP proteins can promote phenotypes associated with malignancy, the SASP is complex, and not all components are cancer promoting [1]. It is likely that each SASP factor may have effects that depend on the cell and tissue context [25]. Some SASP components are chemoattractive factors that mediate the clearance of senescent cells *in vivo* through attracting cells of the immune system [16, 18]. In this context it is conceivable that recruitment of immune cells and the outcome of the immune response might be dependent on the composition of the senescence-associated secretome.

The function of senescence as a barrier to tumor progression and its role in tumor initiation and progression has been demonstrated in murine xenograft models [24, 22, 25]. However, the presence and relevance of senescent tumor cells in untreated human tumors has been little investigated. In the present study we investigated senescence-associated β-galactosidase (SAβ-gal) activity within 129 breast cancer samples from patients who had not been treated with neoadjuvant therapy. We observed differences in the amount of SAβ-gal positive tumor cells between and within breast cancer subtypes. The highest percentage of SAβ-gal positive tumor cells was found in the HER2+ and luminal A breast cancer samples, whereas the vast majority of triple negative tumors displayed either very few or no SAβ-gal positive tumor cells. As breast cancer subtypes differ in their driver mutations, and the induction of senescence can vary in response to differing oncogenic stimuli, the amount of SAβ-gal positive tumor cells likely reflects differences in the ability of breast cancer subtypes to undergo senescence. Alternatively or in addition, the composition of the secretome released by senescent tumor cells from different breast cancer subtypes might be very distinct with respect to their ability to recruit immune cells that eliminate senescent cells, with the consequence that senescent cells might be removed efficiently by the immune system in some breast cancer subtypes, but not in others.

## RESULTS

### Constitution of the patient sample collection

Tumor tissue from 176 patients was collected. All patients were female and only 4 of them had a previous history of early breast cancer. To ensure that senescent cells within the tumors were not induced by previous treatments, such as chemo- or radiotherapy, all samples from neoadjuvantely treated patients were excluded from this study. The ages ranged from 32 to 84 years, with a median of 58.6 years. The other clinicopathological data are summarized in Table 1. Only 129 of the 176 cases were able to be used in this study as described in the Materials and Methods section. The tumor tissue of these 129 patients was classified according to the St. Gallen molecular subtypes. The division of this patient collective into the main molecular breast cancer subtypes is shown in Table 2.

**Table 1:** Relation of histological tumor type, histological grading and pT-stage of all 176 patients

Histological Tumor Type		Histological Grading		pT-Stage	
**Invasive ductal carcinoma**	114 (64.8%)	**G1**	42 (24.1%)	**pT1a-c**	109 (61.9%)
**Invasive lobular carcinoma**	30 (17%)	**G2**	79 (44.4%)	**pT2**	56 (31.8%)
**Invasive mucinous carcinoma**	4 (2.3%)	**G3**	53 (30.4%)	**pT3**	9 (5.1%)
**Invasive cribiform carcinoma**	2 (1.1%)			**pTis**	2 (1.1%)
**Tubular carcinoma**	2 (1.1%)				
**Non invasive carcinoma**	2 (1.1%)				
**Other types of invasive carcinoma**	22 (12.5%)				

**Table 2:** Classification of the 129 patients samples used in this study according to the St. Gallen molecular breast cancer subtypes

Molecular subtype	Luminal	Luminal A	Luminal B	HER2+	ER+/HER2+	ER−/HER2+	Triple neg.	Total cases
Number of cases	95	77	18	16	12	4	18	129
**Number of cases %**	**73.6**	**59.7**	**13.9**	**12.4**	**9.3**	**3.1**	**13.9**	**100**

### SAβ-gal-positive tumor cells exist within invasive breast cancer samples

**Figure 1:**
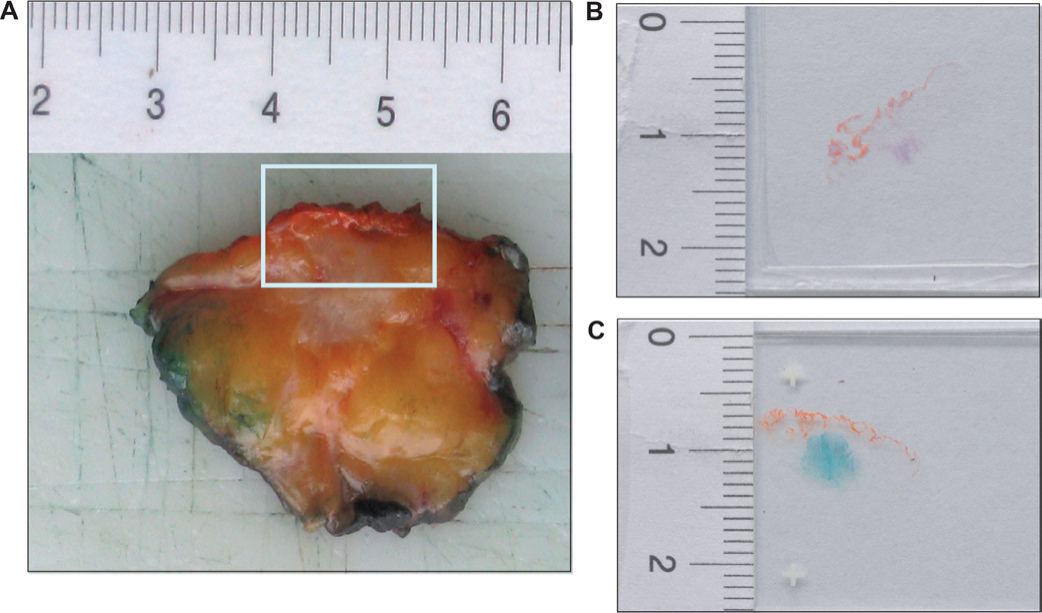
SAβ-gal staining reveals the existence of senescent cells within breast cancer sections (**A**) The frozen tissue of human breast tumours used in this study was collected immediately after an intraoperative diagnostic evaluation. Margins of the surgical specimen were marked with ink. Tumor with the next surgical margin was frozen. In this figure the next surgical margin was marked with orange ink. Two successive frozen sections were generated from tumor tissue immediately after surgury. (**B**) One frozen section was stained with eosin and hematoxilin (H&E), using standard laboratory procedures, and intraoperative examined. (**C**) A second proximate section of the frozen breast tissue from each case was obtained and collected for detection of SAβ-gal activity. SAβ-gal staining reveals the existence of senescent cells within breast cancer sections.

**Figure 2:**
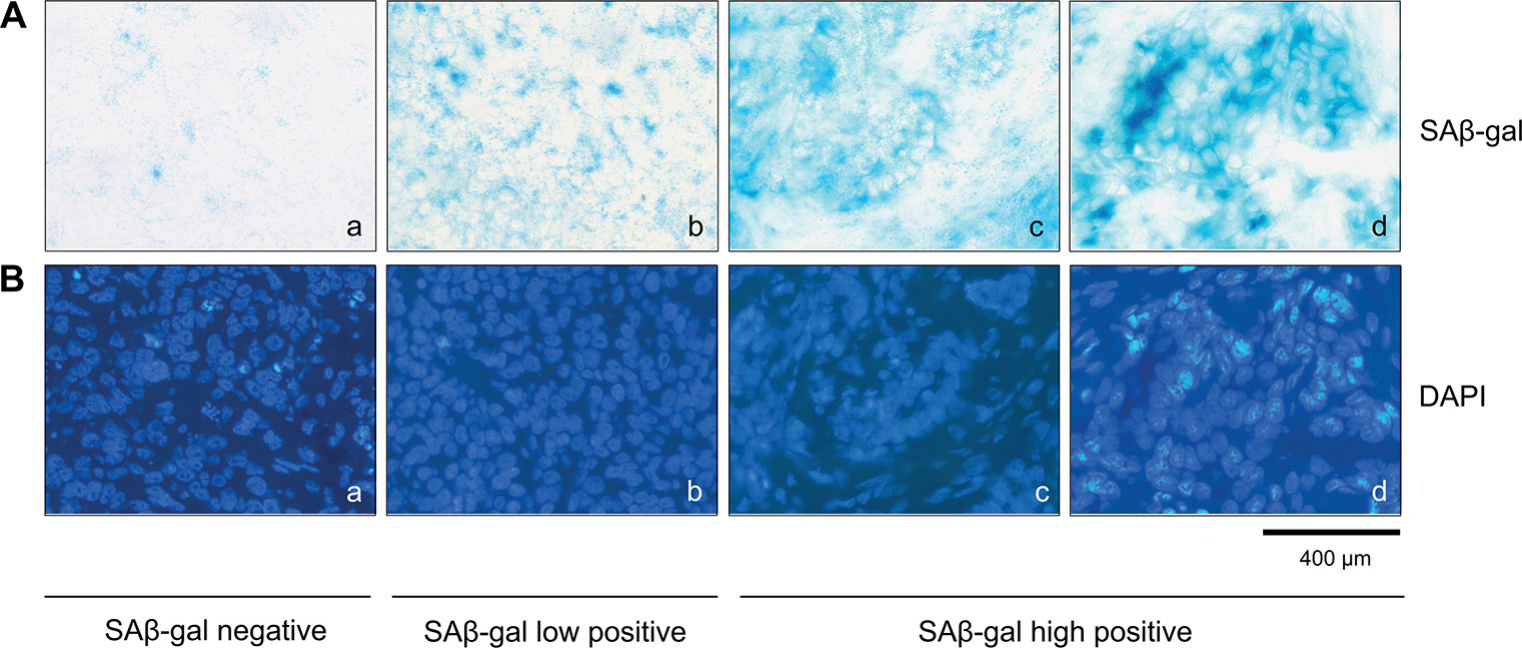
SAβ-gal positive tumor cells exist within invasive breast cancer samples (**A**) SAβ-gal staining of the frozen human breast tumors sections, showing SAβ-gal positive cells (**a**–**d**). Representative examples showing differences in the number of SAβ-gal positive tumor cells in the different samples analysed. (a) SAβ-gal negative; (b) SAβ-gal low positive; (c) and (d) SAβ-gal high positive. (**B**) (a–d) DAPI staining of sections shown in A (a–d). Bars: 400 μm.

Two consecutive frozen sections were generated from the human breast tumor tissue immediately after surgury (Figure 1). One section was used for intraoperative examination and diagnostic evaluation, whereas the second serial section was used for detection of SAβ-gal activity (Figure 1B–1C). Homogeneous cytoplasmic staining with differences in the amount and intensity of SAβ-gal staining was observed within the breast tumor samples (Figure 2A a–d). The vast majority of SAβ-gal positive cells could be clearly identified as tumor cells. However in some samples we were unable to observe SAβ-gal positive tumor cells (Figure 2A a). These tumor samples together with samples in which only non-tumor cells were SAβ-gal positive were defined as SAβ-gal negative. Patient samples in which marginal cytoplasmatic staining (< 50% of tumor cells) was observed were designated as SAβ-gal low (Figure 2A b). Samples with strong cytoplasmatic staining of tumor cells (> 50% of tumor cells) were defined as SAβ-gal high (Figure 2A c–d).

### Detection of SAβ-gal positive cells distinguishes different molecular breast cancer subtypes

**Figure 3:**
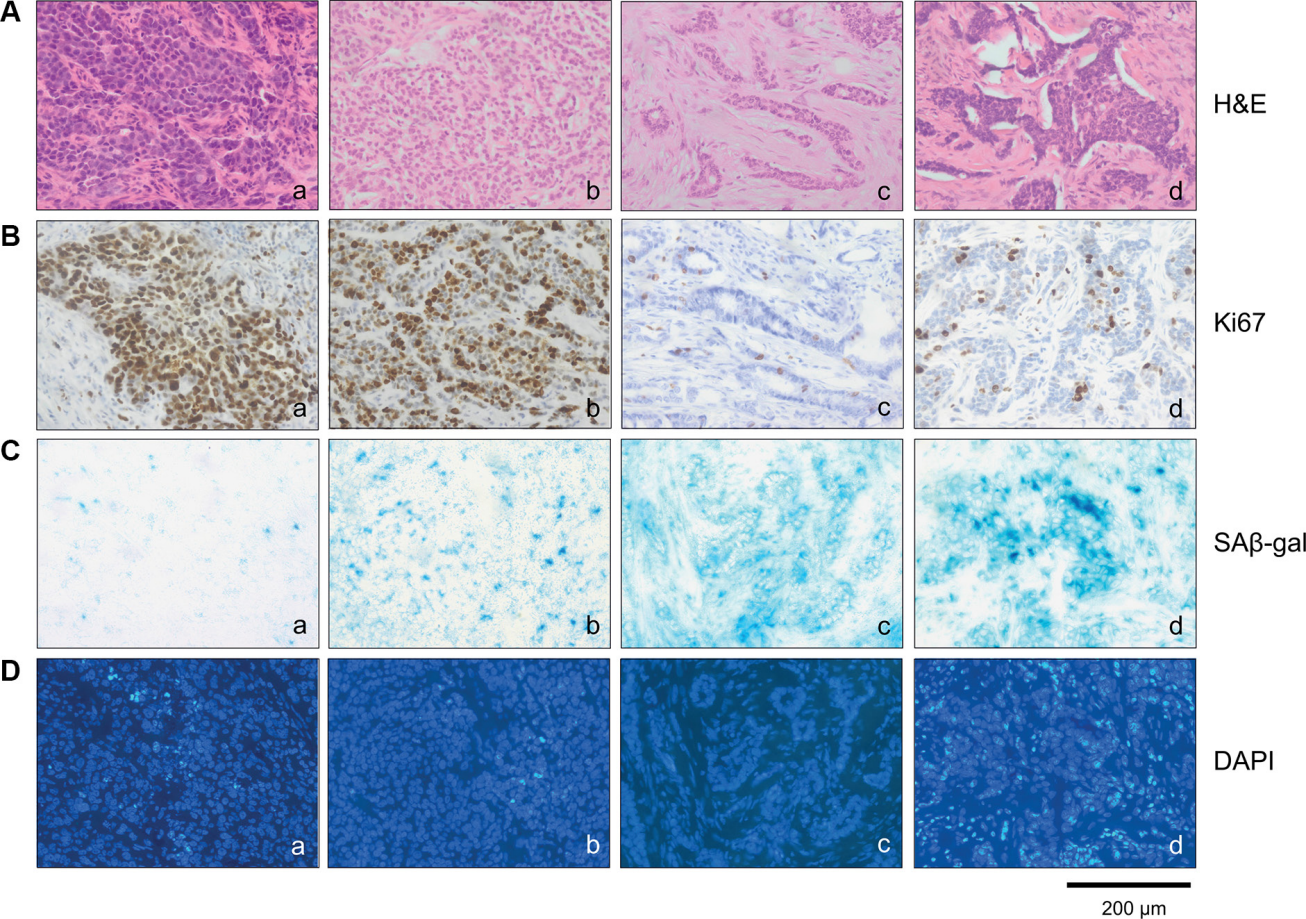
SAβ-gal positive cells distinguish different molecular breast cancer subtypes Tumor sections were stained with (**A**) (a–d) Hematoxilin and eosin, (**B**) (a–d) Ki67 antibodies, (**C**) (a–d) SAβ-gal expression and (**D**) (a–d) DAPI. Represent examples of the different breast cancer subtypes are shown. a) TNBC; SAβ-gal negative and Ki67 > 40% b) Luminal B; SAβ-gal low positive and Ki67 > 40% c) Luminal A; SAβ-gal high positive and Ki67 < 40% d) HER2 positive; SAβ-gal high positive and Ki67 < 40%. Bars: 200 μm.

Next we investigated whether the appearance of SAβ-gal positive tumor cells correlates with hormone receptor expression and HER2 status. Interestingly, we observed the highest percentage of high SAβ-gal positive tumor cells within HER2 positive patient samples (87.5%) whereas the vast majority of triple negative breast cancer (TNBC) samples did not display any SAβ-gal positive tumor cells (88.9%) (Table 3) (Figure 3C a–d). Luminal breast cancers also displayed high percentages of strongly SAβ-gal positive tumor cells (72.6%). However, differences were seen in the percentages of high, low SAβ-gal positive and SAβ-gal negative tumor cells between luminal A and luminal B tumors (Table 3) and between HER2+/ER+ and HER2+/ER− tumors (Table 3). Statistical analysis identified significant differences in the distribution of SAβ-gal positive and SAβ-gal negative tumor cells and in the distribution of SAβ-gal positive and SAβ-gal low/negative tumor cells among the molecular breast cancer subtypes (Table 4).

**Table 3:** Quantification of SAβ-gal positive cells reveals different distribution patterns of SAβ-gal positive cells within molecular breast cancer subtypes

Molecular subtype	Luminal	Luminal A	Luminal B	HER2+	ER+/HER2+	ER−/HER2+	Triple neg.	Total cases
Number of cases	**95**	77	18	**16**	12	4	**18**	129
**SAβ-gal high**	**69 (72.6%)**	58 (75.3%)	11 (61.1%)	**14 (87.5%)**	11 (91.7%)	3 (75%)	**0 (0%)**	83 (64.3%)
**SAβ-gal low**	**22 (23.1%)**	18 (23.4%)	4 (22.2%)	**1 (6.3%)**	1 (8.3%)	0 (0%)	**2 (11.1%)**	25 (19.4%)
**SAβ-gal negative**	**4 (4.2%)**	1 (1.3%)	3 (16.7%)	**1(6.3%)**	0 (0%)	1 (25%)	**16 (88.9%)**	21 (16.3%)

**Table 4:** Significant differences in the distribution of SAβ-gal positive versus SAβ-gal low + negative and SAβ-gal positive versus SAβ-gal negative tumor cells between breast cancer subtypes

	SAβ-gal	Cases	A	B	C	D
**Luminal A+B**	high	69	69 (72.6%)	69 (72.6%)		
	low	22	26 (27.4%)			
	negative	4		4 (4.2%)		
**Triple negative**	high	0	0 (0%)	0 (0%)	0 (0%)	0 (0%)
	low	2	18 (100%)		18 (100%)	
	negative	16		16 (88.9%)		16 (88.9%)
**HER2+**	high	14			14 (87.5%)	14 (87.5%)
	low	1			2 (12.5%)	
	negative	1				1 (6.3%)
**Statistical significance**			***p* < 0.001**	***p* < 0.001**	***p* < 0.001**	***p* < 0.001**

### SAβ-gal positive tumor cells show inverse correlation with the proliferation marker Ki67

Senescence cells are typified by cell cycle arrest and therefore SAβ-gal-positive senescent cells should not proliferate. To demonstrate that SAβ-gal positive cells are growth arrested, we aimed to analyse expression of the proliferation marker Ki67 within SAβ-gal positive tumor cells. Double staining of immunohistochemical and SAβ-gal staining was not possible due to incompatibility of the fixations required for the two stains. Therefore, to assess the proliferation status of SAβ-gal positive tumor cells, we first quantified the percentages of Ki67 positive tumor cells within all 129 tumors using paraffin sections from a piece of tumor that corresponded exactly with the position in the tumor from which the frozen sections for SAβ-gal staining had been taken. The percentages of Ki67 positive tumor cells within all 129 patient samples were in a range from approximately 5 to 90%. Separating the tumors into Ki67 low (Ki67 < 40%) or high (Ki67 > 40%) revealed that the majority of the luminal and HER2 amplified patient samples had less then 40% Ki67 (range 5–35%), whereas approximately 90% of all triple negative patient samples had more than 40% Ki67 (range 40–90%) (Table 5). Patient samples that displayed high percentages of high SAβ-gal positive cells had correspondingly low percentages of Ki67 positive cancer cells, and vice versa (Table 6). Statistical analysis revealed a statistically significant inverse correlation between SAβ-gal positivity and Ki67 positivity, supporting the notion that senscent SAβ-gal-positive tumor cells are cell cycle arrested.

**Table 5:** Distribution of Ki67 positive cells among breast cancer subtypes

Molecular subtype	Luminal	Luminal A	Luminal B	HER2+	ER+/HER2+	ER−/HER2+	Triple neg.	Total cases
Number of cases	**95**	77	18	**16**	12	4	**18**	129
**< 40% Ki67**	**89 (93.7%)**	77 (100%)	12 (66.7%)	**12 (75%)**	10 (83.3%)	2 (50%)	**2 (11.1%)**	103 (79.8%)
**≥ 40% Ki67**	**6 (6.3%)**	0 (0%)	6 (33.3%)	**4 (25%)**	2 (16.7%)	2 (50%)	**16 (88.9%)**	26 (20.1%)

**Table 6:** Significant inverse correlation between SAβ-gal positivity and Ki67 expression

	SAβ-gal	Cases	A	B	C
**< 40% Ki67**	high	76	76 (73.8%)	76 (73.8%)	76 (73.8%)
	low	24	27 (26.2%)		24 (23.3%)
	negative	3		3 (2.9%)	
**≥ 40% Ki67**	high	7	7 (26.9%)	7 (26.9%)	7 (26.9%)
	low	4	19 (73.1%)		4 (15.4%)
	negative	15		15 (57.9%)	
**Statistical significance**			***p* < 0.001**	***p* < 0.001**	***p* = 0.4**

### SAβ-gal positivity inversely correlates with expression of the nuclear lamina protein Lamin B1

**Figure 4:**
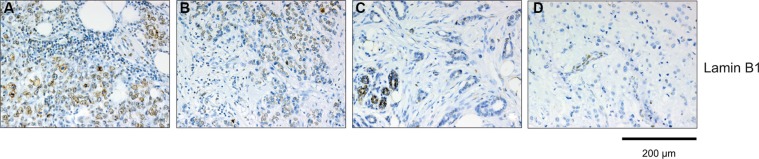
Immunohistochemical analysis of Lamin B1 expression within breast cancer subtypes Tumor section were stained anti-Lamin B1 antibodies. Represent examples of the different breast cancer subtypes are shown: (**A**) TNBC, Lamin B1 high; (**B**) Luminal B, Lamin B1 high; (**C**) Luminal A, Lamin B1 low; (**D**) HER2 positive, Lamin B1 low. Luminal cells within normal ducts in sections of Luminal A and HER2 positive tumors are Lamin B1 positive and serve as internal positive control. The sections used for Lamin B1 expression are serial sections from the same tumor samples used in Figure 3. Bars: 200 μm.

Although SAβ-gal expression is the most widely used marker to detect cellular senescence *in vitro* and *in vivo*, SAβ-gal positivity alone is not always a reliable indicator of whether cells are in the senescent state. Therefore we investigated whether SAβ-gal expression correlates with loss of the nuclear lamina protein Lamin B1, which also serves as biomarker for senescent cell [26–28]. As patient samples from triple negative and HER2+ breast tumors displayed the largest differences between high SAβ-gal positive tumor cells (Table 3; Table 4; Figure 3), these samples were chosen to investigate potential differences in Lamin B1 expression. Immunohistochemical analysis revealed that SAβ-gal positivity inversely correlated with Lamin B1 expression in these samples (Figure 3; Figure 4; Table 7), supporting the notion that SAβ-gal-positive tumor cells are indeed in the senescent state. Consistently, representative sections of luminal A tumors with a high proportion of SAβ-gal positive cells also displayed no or only low numbers of Lamin B1 positive cells. Notably, normal luminal cells showed high expression of Lamin B1, whereas adjacent tumor cells expressed low or no Lamin B1 (Figure 4C–4D).

**Table 7:** Lamin B1 expression within triple negative and HER2+ breast cancer

Molecular subtype	Cases	%	Molecular subtype	Cases	%
**HER2+**	**16**	**100**	**Triple negative**	**18**	**100**
LaminB1 high	3	18.8	LaminB1 high	15	83.3
LaminB1 low	3	18.8	LaminB1 low	0	0
LaminB1 negative	10	62.5	LaminB1 negative	3	16.7

### Detection of p53, p16^INK4a^ and p21^Cip1/Waf1^ within triple negative and HER2+ breast cancer samples

The ability of cells to induce the senescence program depends pivotally on the tumor suppressor pathways Arf-p53 and pRB-p16^INK4a^ [10, 1]. Loss of p53 or mutations in *TP53* that turn p53 into an oncogene, as well as inhibition or loss of pRB and simultaneous overexpression of p16^INK4a^ are associated with escape from senescence and an aggressive phenotype of breast cancer phenotype [12, 13, 6, 31, 32]. Although p16^INK4a^ is increased in senescent cells and therefore is often used as a senescence marker, however p16^INK4a^ can only stop proliferation of cells with a properly functioning p16^INK4a^-pRB-pathway [32]. Thus, only in cells which still have a functional pRB-p16^INK4a^-pathway increased expression of p16^INK4a^and low Ki67 index can be associated with the outcome of senescence. In contrast increased p16^INK4a^ expression levels associated with high Ki67 expression are often characterized by loss of pRB heterozygosity [32]. Under these conditions p16^INK4a^ is an indicator for loss of pRB function and uncontrolled proliferation rather than a marker of senescence [31, 32]. For this reason we used p16^INK4a^ expression in tumor samples with high percentages of Ki67 positive cells as an indicator of a defective p16^INK4a^-RB-pathway.

**Figure 5:**
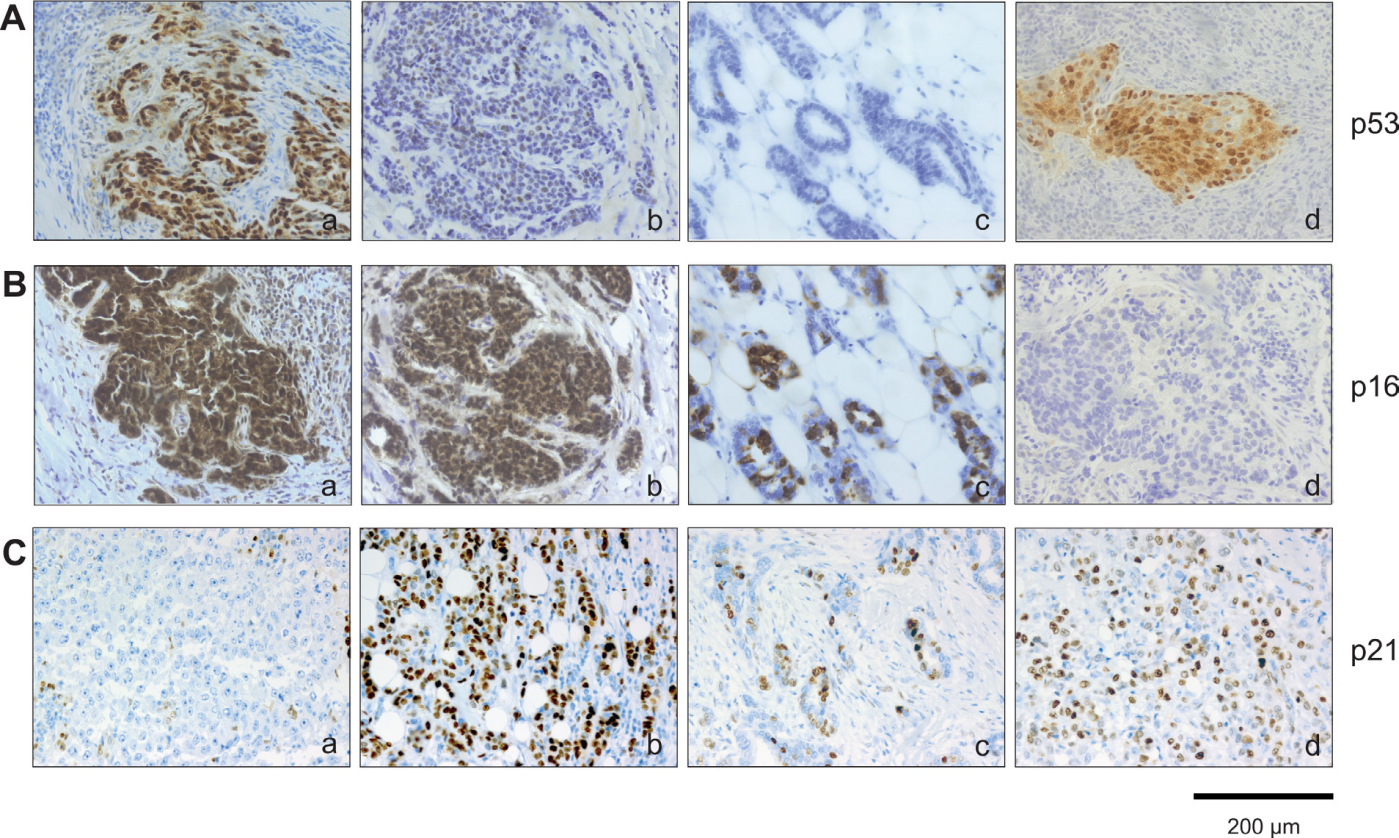
Immunohistochemical analysis of p53, p16^INK4a^ and p21^Cip1/Waf1^ expression within breast cancer subtypes Tumor section were stained (**A**) anti-p53 antibodies and (**B**) anti-p16^INK4a^ antibodies. Represent examples of the different breast cancer subtypes are shown: (A): (a) TNBC, p53 high; (b) Luminal B, p53 low; (c) Luminal A, p53 low; (d) HER2 positive, p53 high. (B): (a) TNBC, p16 high; (b) Luminal B, p16 high; c) Luminal A, p16 high; d) HER2 positive, p16 low. (**C**): (a) TNBC, p21 low; (b) Luminal B, p21 high; (c) Luminal A, p21 high; d) HER2 positive, p21 high. The sections used for p53, p16^INK4a^ and p21^Cip1/Waf1^ expression are serial sections from the same tumor samples used in Figure 3. Bars: 200 μm.

Given the dependence of the senescence program on the tumor suppressor pathways Arf-p53 and pRB-p16^INK4a^ [10, 1], we investigated whether differences in the proportion of SAβ-gal-positive tumor cells within the breast cancer samples correlate with alterations in p53 and p16^INK4a^ expression. To this end, triple negative and HER2+ breast tumor samples (low and high SAβ-gal positivity, respectively (Table 3; Table 4; Figure 3)) were immunohistochemically stained for p53 and p16^INK4a^ expression. Increased expression of p53 and p16^INK4a^ was observed in tumor samples from both subtypes (Table 8; Figure 5), indicating alterations in these two pathways in HER2+ as well as in TNBC samples. However, alterations in both pathways in one and the same sample were only found in TNBC samples (Table 8). We observed high expression of p53 within 55.5% of the TNBC samples, suggesting the presence of *TP53* mutations in these cases (Table 8). In contrast, 61.1% displayed high expression of p16^INK4a^. These findings point to loss or inhibition of pRB and thus impairment of the pRB-p16^INK4a^ tumor suppressor pathway (Table 8). Interestingly, all TNBC samples with high p53 expression also exhibited high p16^INK4a^ expression (Table 8). These observations therefore suggest that 55.5% of the TNBC samples have defects in both the Arf-p53 and the pRB-p16^INK4a^ pathways. Within the HER2+ breast cancer samples we observed high expression of p53 within 31.1% and high p16^INK4a^ expression in 25% of the cases. However, in contrast to the TNBC samples we could not detect simultaneously high p53 and high p16^INK4a^ expression in the HER2+ samples (Table 8).

**Table 8:** Different p53, p16^INK4a^ and p21^Cip1/Waf1^ expression in triple negative and HER2+ breast cancer samples

Molecular subtype	Cases	%	Molecular subtype	Cases	%
**HER2+**	**16**	**100**	**Triple negative**	**18**	**100**
p53 high	5	31.3	p53 high	10	55.5
p53 low	8	50	p53 low	5	27.7
p53 negative	3	18.8	p53 negative	3	16.7
p16 high	4	25	p16 high	11	61.1
p16 low	8	50	p16 low	3	16.7
p16 negative	4	25	p16 negative	4	22.2
**p53 high + p16 high**	**0**	**0**	**p53 high + p16 high**	**10**	**55.5**
p21 high	11	68.8	p21 high	2	11.1
p21 low	3	18.8	p21 low	14	77.7
p21 negative	2	12.5	p21 negative	2	11.1
**p53 neg. + p21 neg.**	**2**	**12.5**	**p53 neg. + p21 neg.**	**2**	**11.1**

The cell cycle inhibitor p21^Cip1/Waf1^ is a p53 target gene [33]. Elevated levels of p21^Cip1/Waf1^ upon p53 activation cause cell cycle arrest and under certain conditions also senescence. Therefore increased p21^Cip1/Waf1^ expression in association with SAβ-gal expression can serve as a senescence marker, and also as a marker for p53 tumor suppressor activity in senescent cells. We therefore investigated next whether differences in the proportion of SAβ-gal-positive tumor cells within the breast cancer samples correlates with p21^Cip1/Waf1^ expression. Immunohistochemical analysis of p21^Cip1/Waf1^ revealed low or even no expression of p21^Cip1/Waf1^ within 77.7% of the TNBC samples (Table 8). In contrast, we observed increased expression of p21^Cip1/Waf1^ within 68.8% of the HER2+ samples (Table 8). These findings suggest that SAβ-gal positivity correlates with high p21^Cip1/Waf1^ levels (Figure 3; Figure 5; Table 8), supporting the notion that SAβ-gal-positive tumor cells are growth arrested and senescent. Interestingly, patient samples that showed no expression of p21^Cip1/Waf1^ were also negative for p53 (Table 8). This observation suggests that loss of p21^Cip1/Waf1^ expression correlates with either inactivation of p53 protein expression or with p53 nonsense mutations that are not detected by the antibodies used in this study.

### Detection of mutations within exons 5, 6, 7 and 8 of *TP53* in triple negative and HER2+ breast cancer samples that express high levels of the p53 protein

The observation that TBNC samples display no or only low numbers of SAβ-gal+ tumor cells but exhibit the highest levels of p53 protein expression in IHC stainings (Figure 3; Table 3; Figure 5) suggests that *TP53* mutations may lead to altered or loss of p53 function in these samples. However, although high p53 levels are suggestive of a mutated *TP53* gene, not all p53 mutations result in protein accumulation. Furthermore, accumulation of wild-type p53 can also occur under certain conditions. We therefore identified *TP53* mutations by genomic sequencing of TNBC and HER2+ samples with the highest p53 protein expression. In total we managed to isolate genomic DNA from eight TNBC and three HER2+ patients and performed sequencing of *TP53* exons 5, 6, 7 and 8 in all of these tumor samples. Exons 5–8 encode the p53 DNA binding domain and contain 80–90% of the *TP53* mutations reported in breast cancer (http://p53.iarc.fr/) [34]. The most frequent substitutions at “hotspot²” codons in exon 5–8 are R248Q, R248W, R175H, R273H, R273C and G245S [34]. These missense mutations lead to loss of p53 wild-type tumor suppressor activity and cause aquired oncogenic potential [34–36]. We found *TP53* mutations in 6/11 patients (Table 9). Notably, two of our TNBC samples had the missense mutations R248Q and R273H (Table 9), which are associated with genomic instability, chemoresistance, reduced apoptosis, increased proliferation rates, cancer-related inflammation and metastasis [36]. These samples are high grade, display no numbers of SAβ-gal positive cells, and contain 80–90% Ki67 positive tumor cells (Table 9). These data support the notion that TNBC samples may contain fewer senescent tumor cells due to alterations in the tumor suppressor functions of p53.

**Table 9:** Mutations within exons 5, 6, 7 and 8 of TP53 in triple negative and HER2+ breast cancer samples that express high levels of the p53 protein

Patient sample	Molecular subtype	Histology	Grading	SAβ-gal	Ki67	p53	p21Cip1/Waf1	p16Ink4A	Exon	Codon Mutation
1.	TNBC	invasive ductal	G3	negative	80%	high	low	high	6	P223L C***C***T→C**T**T
2.	TNBC	invasive ductal	G3	negative	80%	high	low	high	8	R273H C**G**T→C**A**T
3.	TNBC	invasive ductal	G2	negative	40%	high	low	high	-	-
4.	TNBC	invasive ductal	G3	negative	70%	high	low	high	-	-
5.	TNBC	invasive ductal	G3	negative	90%	high	high	high	7	R248Q C**G**G→C**A**G
6.	TNBC	invasive ductal	G3	negative	80%	high	low	negative	5	N131K K132MM133del AAC→AA**G**A***A***G→A**T**GATG→**G**
7.	TNBC	invasive ductal	G3	negative	20%	high	low	low	-	-
8.	TNBC	invasive ductal	G3	negative	90%	high	low	low	5	C176Y T***G***C→T**A**C
9.	HER2+	invasive ductal	G3	negative	60%	high	low	low	-	-
10.	HER2+	invasive ductal	G3	high	40%	high	high	low	8	V272L ***G***TG→**T**TG
11.	HER2+	invasive ductal	G3	high	20%	high	high	negative	-	-

### CD68+ cells exhibit a different distribution within triple negative and HER2+ breast cancer samples

**Figure 6:**
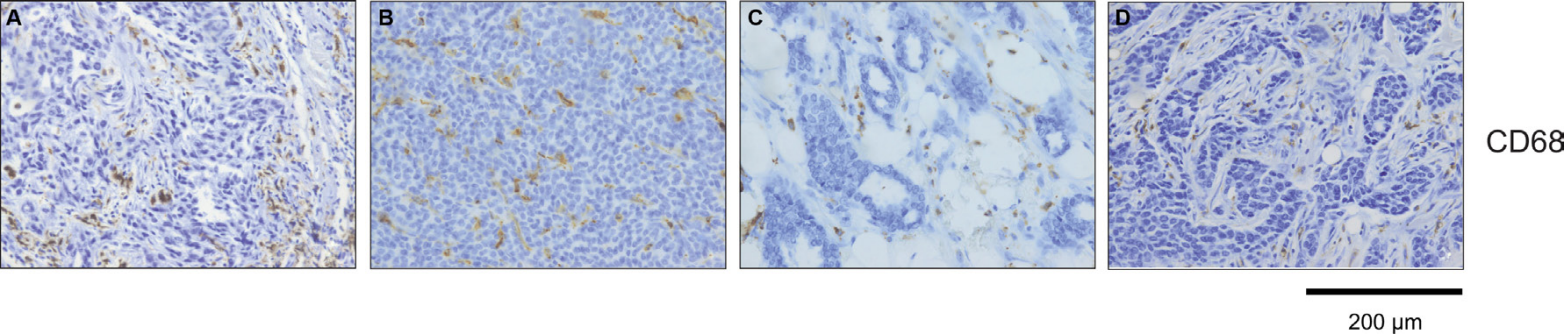
CD68 positive cells are differentially distributed between tumor and stroma within breast cancer subtypes Detection of CD68 positive cells within tumor samples (**A**) TNBC, CD68+ cells within tumor; (**B**) Luminal B, CD68+ cells within tumor; (**C**) Luminal A, CD68+ cells within tumor stroma; (**D**) HER2 positive, CD68+ cells within tumor stroma. Bars: 200 μm.

We next investigated whether senescence in breast cancer subtypes correlates with differences in the recruitment and distribution of specific immune cells as assessed using CD68 immunostaining, a marker for monocytes and macrophages. Interestingly, we observed a different distribution of CD68+ cells within TNBC and HER2+ breast cancer samples (Figure 6A–6D). Specifically, CD68+ cells were mainly located at the stroma of HER2+ samples and were not in contact with tumor cells, whereas CD68+ cells were found between cancer cells in the majority of TNBC samples. Thus it is conceivable that the differences in the number of senescent tumor cells within different breast tumor subtypes might additionally be explained by discrepancies in the clearance of senescent tumor cells.

## DISCUSSION

The notion that cellular senescence is a barrier to oncogene-induced tumorigenesis [10] is supported by studies using human and mouse tissues [4–9]. A limited number of studies have reported that senescent cells exist within premalignant human naevi and colon adenomas, but that during progression to malignant melanomas and adenocarcinomas, senescent cells disappeared or were significantly reduced in number [4, 5], possibly suggesting that cellular senescence may not be a feature of advanced cancers. Our study is the first to date to investigate this issue, and we clearly show that in the context of untreated invasive human breast tumors, significant numbers of senescent cells can be present, in a manner that is dependent on the breast cancer subtype. Specifically we found that high percentages of SAβ-gal positive cancer cells exist within luminal A and HER2+ amplified breast tumors, while few if any SAβ-gal positive cancer cells were observed in TNBC samples. The inverse correlation between SAβ-gal positive and Ki67 positive cancer cells (Table 6) suggests that SAβ-gal positive cancer cells do not proliferate. Loss or decreased amounts of Lamin B1 within the nuclear membrane also correlated with high SAβ-gal positivity, supporting the notion that SAβ-gal positive cells are indeed in the state of senescence [26–28]. As we only included samples in our study from patients who had not received neoadjuvant treatment, we can exclude the possibility that the appearance of senescent tumor cells was the consequence of previous anti-cancer regimens. Therefore, the presence of SAβ-gal positive tumor cells must be caused by either a mechanism intrinsic to the tumor cells themselves or by the microenvironment around them.

The most likely tumor-cell intrinsic mechanism that may underlie the differences in senescene we observed amongst different subtypes of breast cancer are subtype-specific oncogenic stimuli that lead to the initiation and progression of breast cancer. We note that different breast cancer subtypes are typified by distinct subsets of genetic and epigenetic abnormalities [38]. Given the role of senescence as a barrier to oncogene-induced transformation, one explanation of our findings could be that breast cancer subtypes vary in their ability to senesce due to underlying differences in driver mutations and other genetic defects. Another possibility might be that breast cancer subtypes differ in their ability to recruit immune cells that eliminate senescent cells.

The ability of cells to induce the senescence program upon oncogenic stimuli depends mainly on the tumor suppressor pathways Arf-p53 and pRB-p16^INK4a^ [10, 1]. Defects in either one or both of these pathways can circumvent the ability of cells to undergo senescence and increases the susceptibility to cancer progression [6, 11–13, 31, 32]. Although *TP53* mutations are common in human breast cancers and occur in approximately 37% of all cases, there are clear differences between different breast cancer subtypes. Basal-like TNBCs have *TP53* mutations in around 80% of all cases [38]. The majority of *TP53* mutations in TNBCs are nonsense and frame shift mutations leading either to complete loss of p53 tumor suppressor function or gain of oncogenic features. Thus, loss of p53 tumor suppressor function through mutation within TNBCs might be one possible explanation why senescent cancer cells were so few or even absent from the TNBCs in our study. Consistent with this notion, we observed high expression of p53 in 55.5% of our TNBC samples (Table 8) and found mutations in exons 5–8 of *TP53* in 5/8 TNBC samples with the highest p53 protein levels (Table 9). Moreover, 61.1% of the TNBCs displayed high expression of p16^INK4a^ associated with high percentages of Ki67 positive cells (Table 8), indicating impairment of the pRB-p16^INK4a^ tumor suppressor pathway [31, 32]. Interestingly, all TNBC samples with high p53 expression also displayed high p16^INK4a^ expression and 88.9% were either low or negative for p21^Cip1/Waf1^ expression (Table 8). Thus it is likely that over half of the TNBC samples had defects in both Arf-p53 and pRB-p16^INK4a^, providing a further possible reason for why so few senescent cells were detected in the TNBC samples.

Almost all TNBC samples with high p53 protein levels exhibited low numbers of p21^Cip1/Waf1^ positive cells, possibly indicating alterations in p53 tumor suppressor activity (Table 9). Interestingly, only the TNBC patient sample with the missense mutation R248Q showed increased expression of p21^Cip1/Waf1^ (Table 9). This observation might point to a p53-independent mechanism that causes p21^Cip1/Waf1^ overexpression within this tumor sample. Furthermore, it was recently reported that subsets of p53-deficient human cancer cells and tumors exhibit chronic overexpression of nuclear p21^Cip1/Waf1^, which in turn leads to escape from senescence, deregulation of replication licensing, replication stress, genomic instability and chemotherapy resistance [37]. Thus, p21^Cip1/Waf1^ may exert tumor-promoting effects in the context of certain p53 mutations.

Luminal A breast tumors in general have the best prognosis and are those tumors most likely to retain activity of the major tumour suppressor pathways Arf-p53 and pRB-p16^INK4a^ [38]. *TP53* mutations within luminal A and B breast cancers are predominantly missense mutations and do not always inevitably cause complete reduction of p53 tumor suppressor function. They also occur at a much lower frequency than in TNBC, with approximately 12% of luminal A and 29% of luminal B tumors having *TP53* mutations [38]. It is therefore interesting to note that 75.3% of the luminal A samples contained SAβ-gal positive cancer cells, in contrast to 61.1% in the luminal B samples (Table 3). Thus the reduced number and type of *TP53* mutations within luminal A breast tumors in particular is likely to contribute to the larger numbers of senescent cells in this breast cancer subtype.

A large proportion (87.5%) of the HER2+ breast cancer samples in this study contained SAβ-gal-positive cancer cells. At the same time we observed high expression of p53 within 31.1% of the samples (probably indicative of *TP53* mutations in these cases), high p16^INK4a^ expression in 25% and high p21^Cip1/Waf1^ expression in 68.8% of the samples (Table 8). In the HER2+ breast cancer sample in which we found a p53 mutation within codon 272 of exon 8, a high percentage of SAβ-gal positive tumor cells was observed (Table 9), indicating that either the p53 mutation has no consequences, or that HER2+ tumor cells still have the ability to senesce even if p53 is mutated. Collectively these observations suggest that HER2+ tumor cells can senesce even in the presence of a defective Arf-p53 or p16^INK4a^ pathway in at least some of the samples. A possible explanation might be that even if HER2+ breast cancer cells harbour p53 or p16^INK4a^ mutations, they may still be susceptible to induction of senescence. Alternatively or in addition, it has been reported that the secretome of senscent cells transformed by constitutive HER2 signalling inhibits the clearance of senescent cells and exerts prometastatic effects [25]. Thus, the high percentage of SAβ-gal positive cancer cells within our HER2+ breast cancer samples might suggest that oncogenic HER2-induced senescence results in a secretome that can inhibit the recruitment of immune cells and thereby reduced elimination of the senescent cells, leading to accumulation of senescent cells within these tumors. It is also conceivable that the secretome might increase the ability of non-senescent cancer cells to proliferate and metastasize.

The secretome produced by senescent cells can trigger senescence surveillance within liver tumors through the recruitment of macrophages, neutrophils and NK cells [16, 19]. It is therefore of note that immunohistochemical staining for the monocyte/macrophage marker CD68 revealed a breast cancer subtype-specific distribution of macrophages within the 129 breast cancer samples used in this study. In the HER2+ samples, macrophages were mainly located at the stroma and not in contact with tumor cells, while in the majority of TNBC samples, macrophages could be found between cancer cells (Figure 6). This observation suggests that breast tumors subtypes may differ in their SASP and thus in their ability to recruit immune cells to clear senescent cells [16, 18, 19, 25].

The secreome produced by senescent cells can affect the behavior of neighboring cells [1], and may contribute to the ability of senescent cells to promote malignant progression in mice xenograft models [24, 22]. Furthermore, the secretome of senscent cells transformed by constitutive HER2 signalling inhibits the clearance of senescent cells, leading to pro-metastatic effects that can contribute to breast cancer progression [25]. Therefore, it is conceivable that senescent cells within human cancers might contribute to disease progression. Notably, some subpopulations of immune cells can inhibit tumor growth, whereas others promote tumor progression and metastasis [39]. The secretome of senescent cells contains chemokines and other factors that can recruit specific types of immune cell. Thus the exact constituents of the secretome produced by senescent tumor cells may determine whether senescence serves to restrict tumor growth or rather promotes it through the recruitment of tumor-promoting immune cells.

In summary, our observations indicate that senescent tumor cells exist within advanced human breast cancers, and that the proportion of senescent tumor cells varies strongly according to the breast cancer subtype. High percentages of SAβ-gal positive tumor cells exist within luminal A and HER2+ breast cancer samples, whereas no or very few SAβ-gal positive tumor cells are found within TNBCs. One possible explanation for these observations might be that these tumors differ in their genetic and epigenetic alterations, and therefore vary in their capacity to senesce. In addition, clearance of senescent tumor cells may depend on the underlying oncogenes that induce senescence, probably because of differences in their SASP [22, 24, 15, 17]. We suggest that the composition of secretory phenotypes released by senescent tumor cells from different breast cancer subtypes might be very distinct in respect to their ability to recruit immune cells, which can eliminate senescent cells on one hand and regulate tumor growth on the other. Further characterization of the SASPs from different breast cancers subtypes and their potential role in tumor progression is therefore warranted.

## MATERIALS AND METHODS

### Patient samples

Tumor tissue from 176 patients with primary early breast cancer was collected between January 2011 and December 2013 in the Institute of Pathology, Johannes Gutenberg University, Mainz (66 patients in 2011; 78 patients in 2012 and 32 patients in 2013). All patients received a modified radical mastectomy or breast-conserving therapy with sentinel lymph node resection or axillary lymph node dissection. Patients who had been given neoadjuvant therapy were excluded from this study. All included patients had pathological evaluation carried out at the Institute of Pathology. The clinicopathologic data collected included patient age, histological tumour type, TNM classification, histological grade, oestrogen receptor status (ER), progesterone receptor status (PR), HER-2-neu status (HER2) and proliferation index (Ki67). The original pathology reports from all included patients were used. Tumors were staged according to the TNM classification of malignant Tumours (7th Edition 2009), and were classified according to the WHO classification of tumours of the breast [40]. Histological grade was scored according to the Nottingham histologic score system (the Elston-Ellis modification of the Scarff-Bloom-Richardson grading system) [41].

The frozen human breast tumour tissue used in this study was collected immediately after an intraoperative diagnostic evaluation. All cases were previously histologically diagnosed with a preoperative needle core biopsy. The intraoperative assessment served only as delimitation and evaluation of the microscopic tumor margins. For this evaluation the margins of the surgical specimen were marked with ink. Tumor with the nearest surgical margin was frozen (Figure 1A). In Figure 1 the nearest surgical margin was marked with orange ink. One frozen section was obtained, stained with eosin and hematoxylin (H&E) using standard laboratory procedures, and intraoperatively examined (Figure 1B). A second cryosection of the frozen breast tissue from each case was obtained and collected for further analysis (Figure 1C). In only 129 of the 176 cases did the frozen sections contain enough invasive tumor tissue to be included in this study. Two patients had a non-invasive breast cancer. In 12 cases only non-invasive tumour was detected in the cryosection and in 33 cases there was not enough tumor tissue in the cryosection.

### Ethics statement

The use of the patient tissue samples have been conducted in accordance with the ethical standards and according to the Declaration of Helsinki and according to national and international guidelines and have been approved by the authors' local ethical review board.

### Immunohistochemical detection and scoring method

Formalin-fixed, paraffin-embedded tumor sections were used for immunhistochemical detection. Immuno- histochemistry was performed using standard laboratory procedures. Staining was performed on an immunostainer (Techmate 500; Dako, Glostrup, Denkmark) according to the manufacturer's instructions. The antigen-antibody binding was visualised by means of the avidin-biotin complex (ABC method) using AEC (3-amino-9-ethylcarbazol) as chromogen. The primary monoclonal antibodies used in this study were purchased from Dako (Glostrup, Denmark) and were directed agains ER (1D5), PR (1A6), Ki67 antigen (MIB-5), p21^Cip1/Waf1^ (Sx1118) and p53 (DO-7) or Abcam (Cambridge, UK) directed against Lamin B1 (ab16048). The p16^INK4A^ expression was analysed using CINtec^™^ p16 (E6H4) (Roche Diagnostics, Basel, Switzerland). The HER2 status was determined by using the Hercept-test and HER2 FISH pharmDx^™^ Assay Kit (Dako, Glostrup, Denmark).

Ki67 proliferative index, p16^INK4a^, p21^Cip1/Waf1^, p53 and Lamin B1 protein expression were quantified on the basis of percentage positivity in at least 500 neoplastic cells counted in the tumor area. For Ki67 proliferative index, p16^INK4a^, p21^Cip1/Waf1^ and p53 expression only nuclear reactivity and for Lamin B1 only reactivity of the nuclear membrane was taken into account. ER and PR status were determined on the basis of the immunohistochemical intensity and the percentage of positive cells. A strong nuclear staining in one or more tumor cells was considered positive. Complete and strong membrane staining in > 10% of the tumour cells qualified for HER2 overexpression (3+). A HER2:Cep17 ratio > 2.0 was regarded as a HER2 amplified tumor [42].

### St. Gallen risk groups

We classified all cases in accordance with the St. Gallen international breast cancer conference guidelines from 2013 with the suggested definition of intrinsic subtypes of breast cancer: luminal-A (ER+ and PR+, Ki67 low (≤ 20%) and HER2−), luminal-B (ER+ and PR−/low, or Ki67 high (> 20%) and HER2+/−), HER-2 positive (ER−, PR− and HER2+) and triple negative (ER−, PR−, HER2−) [43].

### Senescence-associated β-galactosidase activity

Senescence-associated β-galactosidase (SAβ-gal) activity at pH 6.0, the standard biomarker of cellular senescence, was detected *in vitro* or *in vivo* as described previously [44]. Briefly, frozen sections of human breast tumors were dried over night at room temperature and subsequently fixed with 2% formaldehyde and 0.2% glutaraldehyde for 1 hour at room temperature. After fixation, the sections were incubated in β-galactosidase staining solution [44] for 24 hours at 37C°. Beta-galactosidase was purchased from Carl Roth GmbH, Karlsruhe, Germany. For visualization of the nuclei, sections were incubated in 4′,6-Diamidino-2-phenylindole (DAPI, Sigma Adrich, Taufkirchen Germany).

### Sequencing of genomic DNA from formalin-fixed and paraffin-embedded tumor samples

Tumor areas of serial sections were selectively scratched off according to corresponding HE-slides and, following de-paraffination and Proteinase-K digestion, used for DNA preparation. PCR amplfication was carried out using the primers listed in supplemental information and afterwards the PCR products were Exo-SAP (Affymetrix, Santa Clara, California, USA) digested and sequenced directly using the same primers. At least 2 independent PCR products of each exon 5–8 from each patient were sequenced in two different orientations.

### Statistical analysis

All data were analysed in Microsoft Excel. A Fisher's exact test was performed. A *p-value* of < 0.001 was considered to be statistically significant.

## SUPPLEMENTARY MATERIALS


