# Supplementary material for: Detection of cellular senescence within human invasive breast carcinomas distinguishes different breast tumor subtypes
Source: Oncotarget. 2016 Oct 4;7(46):74846–59. doi: 10.18632/oncotarget.12432 (PMC5342706; doi:10.18632/oncotarget.12432)
Supplement: Supplementary file 1 [file oncotarget-07-74846-s001.pdf]

Detection of cellular senescence within human invasive breast carcinomas distinguishes different breast tumor subtypes

Supplementary Materials

SUPPLEMENTARY METHODS

Primer for *TP53* sequencing:

P53-Exon5-F-1: agacgccaactctctctagc
P53-Exon5-R-1: ctcacaacctccgtcatgtg
P53-Exon5-Fnest.-1 ggaggtgcttacgcatgtttg
P53-Exon5-Rnest.-1 tcatgtgctgtgactgcttgt
P53-Exon5-R-2 gtgaggaatcagaggcctgg
P53-Exon5-Fnest.-2 tttctttgctgccgtsttcc
P53-Exon6-F: agatagcgatggtgagcagc
P53-Exon6-R: taagcagcaggagaaagccc
P53-Exon6-Fn: ccaggcctctgattcctcac
P53-Exon7-F: gcctcccctgcttgcc
P53-Exon7-R: ccagtgtgcagggtggc
P53-Exon7-Fn: ctgcttgccacaggtctcc
P53-Exon7-Rn: caagtggctcctgacctgg
p53-Exon8-F: taggacctgatttccttactgcc
p53-Exon8-R: agtgctaggaaagaggcaag
p53-Exon8-Rn: tgaggcataactgcaccct
